# 

**Published:** 1895-04

**Authors:** 


### CONTENTS.

COMMUNICATIONS.

The Reason Why........................................ 157
The Hypodermic use of Cocaine........................................ 175

MONTHLY DIGEST.

Operative Dentistry........................................ 178

SELECTIONS.

Can Not be Sent by Mail........................................ 174
Care of the Poor........................................ 202
Eye Strain a Cause of Nocturnal Enuresis........................................ 202
Board of Regent's Request........................................ 202
Sudden Suppression........................................ 202
Extirpation of the Stomach of a Cat........................................ 208

EDITORIAL.

The American Medical Association........................................ 203
A Return........................................ 204
Retirement........................................ 204

NOTICES.

Mississippi Valley Dental Association........................................ 205
American Medical Association........................................ 206
Bibliographical........................................ 207

